# A systematic literature review of health consumer attitudes towards secondary use and sharing of health administrative and clinical trial data: a focus on privacy, trust, and transparency

**DOI:** 10.1186/s13643-020-01481-9

**Published:** 2020-10-09

**Authors:** Elizabeth Hutchings, Max Loomes, Phyllis Butow, Frances M. Boyle

**Affiliations:** 1 Northern Clinical School, Faculty of Medicine, University of Sydney, North Sydney, NSW Australia; 2 Department of Psychology, The University of Sydney, Sydney, NSW Australia; 3 Centre for Medical Psychology & Evidence-Based Decision-Making (CeMPED), Sydney, Australia; 4 Psycho-Oncology Co-Operative Research Group (PoCoG), The University of Sydney, Sydney, NSW Australia; 5 Patricia Ritchie Centre for Cancer Care and Research, Mater Hospital, North Sydney, Sydney, Australia

**Keywords:** Data sharing, Attitudes, Privacy, Trust, Transparency

## Abstract

We aimed to synthesise data on issues related to stakeholder perceptions of privacy, trust, and transparency in use of secondary data. A systematic literature review of healthcare consumer attitudes towards the secondary use and sharing of health administrative and clinical trial data was conducted. EMBASE/MEDLINE, Cochrane Library, PubMed, CINAHL, Informit Health Collection, PROSPERO Database of Systematic Reviews, PsycINFO, and ProQuest databases were searched. Eligible articles included those reporting qualitative or quantitative original research and published in English. No restrictions were placed on publication dates, study design or disease setting. One author screened articles for eligibility, and two authors were involved in the full text review process. Data was extracted using a pre-piloted data extraction template by one author and checked by another. Conflicts were resolved by consensus. Quality and bias were assessed using the QualSyst criteria for qualitative and quantitative studies. This paper focuses on a subset of 35 articles identified from the wider search which focus on issues of privacy, trust, and transparency. Studies included a total of 56,365 respondents. Results of this systematic literature review indicate that while respondents identified advantages in sharing health data, concerns relating to trust, transparency, and privacy remain. Organisations collecting health data and those who seek to share data or undertake secondary data analysis should continue to develop trust, transparency, and privacy with healthcare consumers through open dialogue and education. Consideration should be given to these issues at all stages of data collection including the conception, design, and implementation phases. While individuals understand the benefits of health data sharing for research purposes, ensuring a balance between public benefit and individual privacy is essential. Researchers and those undertaking secondary data analysis need to be cognisant of these key issues at all stages of their research. Systematic review registration: PROSPERO registration number CRD42018110559 (update June 2020).

## Background

Healthcare provides an opportune setting for increased data sharing and secondary data analysis. Secondary data analysis of existing data originally collected for other purposes [1] can provide insights into real-world clinical practice [2] and generate new clinical evidence [3]. There are many forms of data collected during an individual’s interactions with health services, including administrative and clinical trial data which are the focus of this review. Administrative data are data originally collected for administrative and billing purposes [4], but have the capacity to be used to identify systemic issues and service gaps and used to inform improved health resourcing. Clinical trials are expensive and take an approximately 17 years to complete, and less than 14% of the evidence is translated into practice [5]. Given the low rates of evidence being translated into practice, it can be suggested that the secondary use of this data has greater importance. The secondary analysis of clinical trial data can further advance the medical community’s understanding of diseases and potentially limit the expenditure of funds on already tested hypotheses.

Increased access to data for secondary use is complex and continues to attract strong debate within the health and scientific communities as well as the general public. While researchers are now being encouraged to increase data accessibility for secondary research [6, 7], a range of stakeholder-perceived barriers and concerns remain, including issues such as trust, transparency, and privacy [8, 9]. Despite the impact of these issues on willingness to share data, there is a lack of synthesis of stakeholder views to guide policy and practice.

This paper presents the results of a subset of articles identified in our systematic literature review and focuses on healthcare consumer concerns relating to privacy, trust, and transparency in the setting health administrative and clinical trial data reuse.

## Methods

This systematic literature review presents the results of a subset of articles identified in a larger review of articles addressing data sharing and was undertaken in accordance with the PRISMA statement for systematic reviews and meta-analysis [10]. The protocol was prospectively registered on PROSPERO (www.crd.york.ac.uk/PROSPERO, CRD42018110559; updated June 2020).

The following databases were searched: EMBASE/ MEDLINE, Cochrane Library, PubMed, CINAHL, Informit Health Collection, PROSPERO Database of Systematic Reviews, PsycINFO, and ProQuest. The search was conducted on 24 June 2020. No date restrictions were placed on the search; key search terms are listed in Table 1.

**Table 1:** Example search strategy

PubMed	
1	((data sharing) OR (data link*) OR (secondary data analysis) OR (data reuse) OR (data mining))
**2**	((real world data) OR (clinical trial) OR (medical record*) OR (patient record*) OR (routine data) OR (administrative data))
**3**	attitud* OR view* OR opinion* OR perspective* OR satisfaction)
**4**	(patient* OR consumer*)
**5**	(doctor* OR clinician OR oncologist OR specialist*)
**6**	(Researcher* OR scientist* OR (data custodian*))
**7**	4 or 5 or 6
**8**	1 and 2 and 3
**9**	1 and 2 and 3 and 7

*Search includes ‘wildcards’ or truncation

Our original goal was to focus on attitudes towards data reuse by breast cancer patients. However, due to a paucity of studies targeting this group, we re-ran the search without this limitation and present the results of all disease settings and noted specific cases where breast cancer or any cancers were included. Breast cancer is a disease that impacts older individuals; therefore, respondents under the age of 18 years were excluded from this analysis, as were attitudes towards biobanking and genetic research.

We noted that increasingly the delineation between data collected for administrative purposes and other forms of electronic documentation such as electronic health records (EHR) (or other terms for these) becomes less clear. These records can contain both administrative and clinical data. Where possible, EHRs were excluded from this literature review; however, we acknowledge that the lack of separation has made this a grey area.

Papers were considered eligible if they were published in English in a peer-reviewed journal; reported original research, either qualitative or quantitative with any study design, related to data sharing in any disease setting; and included subjects over 18 years of age. Reference list and hand searching was undertaken to identify additional papers. Systematic literature reviews were included in the wider search but were not included in the results. Papers were considered ineligible if they focused on electronic health records (including other terms for these), health information exchanges, biobanking and genetics, and were review articles, opinion pieces, articles, letters, editorials or non-peer-reviewed theses from masters and doctoral research. Duplicates were removed and title and abstract and full text screening were undertaken using the Cochrane systematic literature review programme ‘Covidence’ [11]. One author screened articles for eligibility and two authors were involved in the full text review process; conflicts were resolved by consensus.

Quality and bias were assessed at a study level using the QualSyst system for quantitative and qualitative studies as described by Kmet et al [12]; this is a validated tool and can be used to assess both qualitative and quantitative studies. No modifications were made to the QualSyst criteria prior to use. Quality and bias assessment was undertaken independently by two authors; conflicts were resolved by consensus. A maximum score of 20 is assigned to articles of high quality and low bias; the final QualSyst score is a proportion of the total, with a possible score ranging from 0.0 to 1.0 [12].

Data extraction was undertaken by one author using a pre-piloted form in Microsoft Office Excel; a second author confirmed the data extraction. Conflicts were resolved by consensus. Data points included author, country and year of study, study design and methodology, health setting, and key themes and results. Where available, detailed information on research participants was extracted including age, sex, employment status, highest level of education, and health status.

Quantitative data were summarised using descriptive statistics. Synthesis of qualitative findings used a meta-aggregative approach, in accordance with guidelines from Lockwood et al [13]. The main themes of each qualitative study were first identified and then combined, if relevant, into categories of commonality. Using a constant comparative approach, higher-order themes and subthemes were developed. Quantitative data relevant to each theme were then incorporated. Using a framework analysis approach as described by Gale et al [14], the perspectives of different groups towards data sharing were identified. Where differences occurred, they are highlighted in the results. Similarly, where systematic differences according to other characteristics (such as age or sex) occurred, these are highlighted.

## Results

**Fig. 1:**
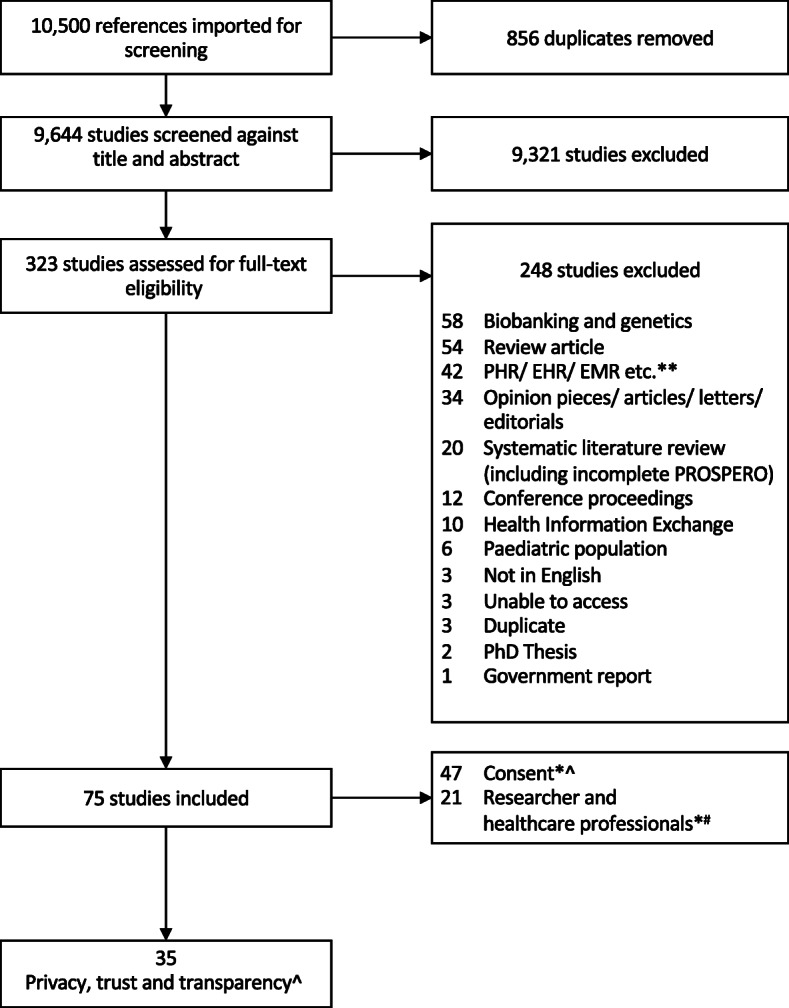
PRISMA flow diagram

This search identified 10,499 articles, of which 323 underwent full text screening; 75 articles met the inclusion criteria for the larger review. The PRISMA diagram is presented in Fig. 1. This article presents a subset of the results of the wider search which explores attitudes of health consumers towards privacy, trust, and transparency. The results relating to attitudes towards data sharing and reuse by researchers and healthcare professionals, and attitudes towards consent in the context of data sharing and reuse by healthcare consumers are presented in subsequent publications.

A subset of 35 [15–49] of the 75 articles addressed issues relating to privacy [15–49], trust [16–18, 21, 23, 24, 26, 28–30, 32–37, 39–42, 44–46, 48], and/or transparency [15–17, 26, 30, 32, 33, 37, 40, 42–44, 48] and are included in this analysis (Fig. 1 and Table 2). A total of 56,365 respondents were included in the studies.

**Table 2:** Included studies

Author, location, data collection date	Methodology, sampling, analysis	Health condition/setting	No. of participants (***N***)	Participant demographics ***n*** (%)	Key themes (alphabetical)	Outcomes, result(s)	QualSyst Score
**Qualitative**							
[15], Britain, October to December 2004	Questionnaire, results expressed in percentages	Recently discharged medical and surgical patients / hospital	166	Not reported	Privacy Transparency	***Privacy*** • UK law currently allow for data use if the data is necessary and the risk to privacy is proportionate. ***Transparency*** • Public education on the secondary use of data promotes transparency.	0.9
[16], Europe, March to May 2018	Survey, descriptive statistics, and chi-square test for independence	Rare diseases/ patients and their families with rare diseases	2013	**Age, years** 2 (0), 15–17 70 (3), 18–24 293 (15), 25–34 852 (42), 35–49 644 (32), 50–64 152 (8), ≥65 **Sex, male** 473 (23) **Location** 1775 (88), EU 238 (12), non-EU **Diagnostic status** 1909 (95), diagnosed 104 (5), undiagnosed **Number of rare disease (*n*** **= 1909)** 1664 (87), 1 174 (9), 2 44 (2), 3 13 (1), 4 14 (1), ≥5	Privacy Transparency Trust	***Privacy*** Willingness to share data is underscored by the need to respect the individual’s privacy wishes and choices regarding the use of their data. ***Transparency*** Improved communication will increase transparency in research, particularly in the setting of shared data. ***Trust*** • Not-for-profit stakeholders (89% for medical doctors, 79% researchers from non-profit organisations, 77% for patient organisations, 69% for healthcare professionals other than medical doctors) is considerably higher than trust in for-profit stakeholders. • Participants with higher levels of education tend to trust government and institutions from their country more (53%) than those who finished school earlier (44% among those who finished school before 20 years old). *Distrust* • 45% are in favour and 50% are opposed to sharing data with researchers from the pharmaceutical industry. • 16% are in favour and 80% are opposed data with insurance companies Older respondents are less likely to trust the private sector: 57% of respondents under 25 trust researchers from pharmaceutical industry compared to 36% for respondents over 65 and only 28% compared to 9% for insurance companies.	0.95
[17], Europe, 2012	Questionnaire, results expressed in percentages	Leukodystrophy/ Leukodystrophy patients and family	195 (149 family, 46 patients)	**Age, years** 121 (62), 40–64 **Country** 130 (66.7), France 24 (12.3), Italy 9 (4.6), Belgium 6 (3.1), Spain 26 (13.3), Germany	Privacy Transparency Trust	***Privacy*** • Ethics committees protect ‘patients’ rights and privacy’. ***Transparency*** • Information and transparency are needed on database governance. • Patient organisations should help with the development of clinical trials. • Transparency needed on the use of data, data storage and the length of data accessibility. • Information on secondary use should be provided at initial consent. • Pharmaceutical industry access to data was supported by some, if transparent. ***Trust*** • High level of trust in researchers using the database. • Patient’s/families had great hope and trust in the development of database research.	0.85
[18], Finland, not reported	Survey, descriptive statistics	General medical/general public	418	**Age, years** 44 (10.5), ≤30 47 (11.2), 31–40 57 (13.6), 41–50 88 (21.1), 51–60 126 (30.1), 61–70 49 (11.7), >70 7 (1.7), missing **Education** 74 (17.7), PS 44 (10.5), SS 165 (39.5), HS 80 (19.1), University of Applied Sciences or bachelor’s degree 52 (12.4), Master’s degree or higher 3 (0.7), missing	Privacy Trust	***Privacy*** • Privacy protection was the most common concern for register-based research. • 80% considered protection of their health information important or very important. 56% had positive attitude towards their own information being used for research. • Sex, education, and health status did not affect this view significantly. • Studies of major public health impact are more important than individual privacy protection—25% somewhat disagreed and 12% strongly disagreed. • Respondents in good health more likely to agree than those with poor health. • >70 years less concerned with privacy than younger age groups; 92% (31–40 years) stated privacy of health information was very important or important. ***Trust*** • 56% had a positive or very positive attitude about their information being used research purposes, 31% were neutral suggesting trust in research organisations and data protection practices. • Younger participants, despite higher concern for privacy, trust researchers and were willing to let their information be used for research.	0.85
[19], Japan, October to November 1995	Survey, Fisher’s exact test and Mantel-Haenszel chi-square	Cancer/outpatients, and inpatients	293	**Age, years** 11 (3.8), ≤29 30 (10.2), 30–39 49 (16.7), 40–49 71, (24.2), 50–59 81 (27.6), 60–69 48 (16.4), ≥70 **Sex, male** 115 (39)	Privacy	***Privacy*** • 82.6% did not mind their own clinical data being used for healthcare and treatment skills improvement under the condition that privacy would not be violated. • Younger males seemed most positive to the use of data to improve healthcare and treatment skills. • No significant difference in responses among three sources of patients.	0.5
[20], Canada, August 2014 and May 2015	Survey, descriptive statistics and Student’s *t* tests and nonparametric tests	Cancer/outpatient clinic	569	**Age, years** 59, median **Cancer type** 109 (19.2), breast 86 (15.1), gastrointestinal 83 (14.6) genitourinary 70 (12.3), thoracic 73 (12.8), hematologic 73 (12.8), head and neck 63 (11.1), gynaecologic 12 (2.1), other **Clinical trial participation, yes** 183 (32.2) **Education** 346 (60.8), university, college, professional 39 (6.9), vocational, technical, diploma 169 (29.7), elementary, HS 15 (2.6), prefer not to answer or missing **Sex, male** 234 (41)	Privacy	***Privacy*** • Willingness to provide health information for research. • Cultural differences between large cities and smaller communities may alter an individual’s level of concern about privacy and confidentiality. • Linkage of clinical trial data with administrative databases requires consideration of ethics and regulatory principles and processes to ensure privacy.	0.75
[21], USA, not reported	Structured survey, univariate statistics, logistic regression models	Clinical trial data /interventional clinical trial participants	771	**Age, years (*n*** **= 762)** 63 (8.3), <25 177 (23.2), 25–44 286 (37.5), 45–64 236 (31), ≥65 **Sex, male** 382 (51.1) **Ethnicity (*n*** **= 768)** 518 (67.4), white 113 (14.7), Black or African American 51 (6.6), American Indian or Alaskan Native 25 (3.3), Asian 61 (7.9), Other **Education (*n*** **= 752)** 40 (5.3), < HS 125 (16.6), HS 206 (27.4) some college 238 (31.6), college 143 (19), graduate degree	Privacy Trust	***Privacy*** 93% of respondents were very or somewhat likely to allow their data to be shared with university researchers 82% were very or somewhat likely to share their data with researchers from for-profit companies. • This willingness did not change of the purpose for the use of data, except for use in litigation. Provided adequate safeguards were in place, most were willing to share their data for research. ***Trust*** • Some believed that sharing this data may reduce participation rates in clinical research (37%), were concerned that it would be used for marketing purposes (34%), stolen (30%), used to discriminate (22%), or for profit (20%). • Respondents had less trust in pharmaceutical companies (18% trusted a great deal or a lot) and insurance companies (15%) compared to universities (63%). • 8% were unwilling to share with for-profit companies, 92% would be very or somewhat willing to share with these companies. • Multivariable modelling found that those who believed that the negative aspects of data sharing outweighed the positives, was significantly higher in those who felt that other people could generally not be trusted, those concerned about being re-identified, or those concerned about information theft. • Those with a college degree were also associated with feeling the negative aspects outweighed the benefits. • Low levels of trust resulted in less willingness to share data with researchers from both not-for-profit and for-profit companies.	0.9
[22], Hong Kong, not reported	RCT nested within a cohort, chi-square test, multivariable logistic regression, likelihood ratio test	General medical/subsample of the FAMILY cohort	1200	**Age, years** 94 (7.8), 18–29 197 (16.4), 30–44 423 (35.3), 45–59 307 (25.6), 60–74 179 (14.9), ≥75 **Education** 436 (36.3), primary 587 (48.9), secondary 177 (14.8), tertiary **Sex, male** 456 (38)	Privacy	***Privacy*** • Requesting HKID significantly reduced consent for data linkage among adults aged 18–44 years (9.9%) compared to other age groups reflecting privacy concerns. • 60–74 years were most likely to agree to data linkage (40.7%).	0.95
[23], Canada, November 2003	Survey, descriptive statistics, multiple logistic regression	AIDS, MS, mental health / outpatients	235	**Age, years** 68 (28.9), 20–39 129 (54.9), 40–59 34 (14.5), ≥60 4 (1.7), unknown **Education** 39 (16.6), <grade 12 35 (14.9), grade 12 156 (66.4), attended/ finished post-secondary 5 (2.1), no answer **Previous experience with medical research, yes** 127 (54) **Sex, male** 86 (36.6)	Privacy Trust	***Privacy*** • Privacy of health and financial records of most concern to respondents. • To maintain privacy, consent to access medical records should be sought. • Concerns included harm due to others accessing record; information being available to ‘many people’. • Privacy and autonomy are of importance. • There was not a willingness to trade loss of privacy for public good. ***Trust*** • Easier to trust researchers when the individual is involved and meets someone involved in the research. • Education is needed to foster trust in health research processes and to provide more information on societal perspectives of personal health data.	0.85
[24], USA, August to November 2014	Health Information National Trends Survey (HINTS), multivariate regression analyses	General medical/general public	3212	**Age, years, mean** 53.48 **Sex, male** 1317 (41)	Privacy Trust	***Privacy*** • Three key facets of health privacy: capital awareness of privacy, attitude towards the importance of privacy and data sharing, and confidence in the ability to maintain privacy. • Positive relationship between privacy capital and engagement. • Development of health privacy capital is susceptible to sociodemographic disparities. • Higher levels of education and income associated with higher levels of health privacy capital. • Awareness and confidence were also related to the social capital index. • Females and older people were more likely to be aware of health information. • Those with higher levels of health privacy capital were more likely to report higher levels of digital interaction with health professionals. • Higher levels of income were found to do consistently better in health outcomes, and they tended to be more actively engaged in digital interaction with health professionals. ***Trust*** • Privacy awareness as a predictor seems to play an enabling role for those who lack basic social networks because it helps them fully trust and efficiently navigate the health system to their benefit. • Promoting trust in health service is important.	0.9
[25], Europe, August to November 2013	Survey, descriptive analysis and choice modelling analysis, Westin’s methodology	General medical/general public	20,882	**Age, years** 2163 (10.4), 18–24 3512 (16.8), 25–34 3719 (17.8), 35–44 3763 (18), 45–54 3735 (17.9), 55–64 3990 (19.1), ≥65 **Health status, self-rated** 12,823 (61.4), good or very good **Sex, male** 9960 (47.7)	Privacy	***Privacy*** • 48.9% to 60.6% of respondents expressed concerns about different levels of access to health data. • 38.4% of respondents agreed that healthcare providers are currently successful in providing effective data security. Sweden, Slovenia, and Denmark had the highest proportions respondents not concerned about privacy; highest conference was in Lithuanian respondents. • Anonymised sharing of information with academic researchers was not preferred, thus pointing towards a preference for individual-level benefits over broader population-level benefits. • Respondents were averse to sharing health information with third parties, such as insurance providers and pharmaceutical companies.	1
[26], Northern Ireland, September to December 2015	NILT survey, univariate and multivariate analyses	General medical/general public	1202	*Results of weighted demographics* **Age, years** 144 (12), 18–24 175 (14.6), 25–34 172 (14.3), 35–44 214 (17.8), 45–54 180 (15.0), 55–64 310 (25.8), ≥65 7 (0.6), not answered/ refused **Education** 224 (18.6), no qualification 555 (46.2), school level 369 (30.7), graduate level 54 (4.5), not answered/ refused **Sex, male** 559 (46.5)	Privacy Transparency Trust	***Privacy*** • 85% agreed data should be used for research which benefits society if anonymous and privacy were maintained. • 95% in favour of sharing data within the health service, over two thirds were in favour of health information being shared to improve access to services provided by other government departments. • Not willing to share data with ‘just anyone’: • 16% happy for university researchers to use data; 39% did not want commercial access to their data; 20% did not care who used their data • 74% did not mind how their data was used if anonymised; 46% believed that even if the data were de-identified you can still identify the person. • 83% believed that the right to privacy should be respected over everything else. • A significant minority showed limited support for data sharing. ***Transparency*** • Minimum requirements for trust: transparency, knowledge about what information is held and what it is used for, and data security. • Transparent communication should help improve trust. ***Trust*** Trust in keep information secure and use appropriately: • 91% GP surgery; 86% NHS; 73% government departments; 72% academic researchers; 51% charities; 41% commercial organisations. • 5% of respondents did not trust any of the organisations. • 42% had ever had concerns about how any of those organisations used the information they kept. • Low levels of trust were associated with a need for consent to use data. • Support for data sharing based on: trust in organisations, data protection measures, and public benefit. Levels of trust differed between: • Sex – females – generally more trusting of organisations. • Age – ≥55 years less trusting of commercial organisations while 25–44 years were more trusting of academic researchers. • Trust included the ability to keep data secure, accurate records, capacity to change or delete incorrect data, data used to discriminate against individual. • 50% believed that commercial organisations should be required to provide more data safeguards, compared to other organisations.	0.9
[27], United Kingdom, 2009	BHPS Wave 18, multivariate bivariate probit models	General medicine/BHPS Wave 18 participants	6433	Not reported	Privacy	***Privacy*** • Attitudes to privacy are related to views on consenting to data linkage. • Privacy concerns were the strongest negative predictor for consent. ***Trust*** • General trust in others positively affect consent.	0.65
[28], New Zealand, not reported	Survey, chi-square tests	General medical/general public	203	**Age, years** 106 (56), 18–34 69 (37), 31–60 14 (7), ≥61 **Sex, male** 61 (32)	Privacy Trust	***Privacy*** • Respondents more willing to share health information if identity removed. • 60% concerned about sharing even anonymous information with people other than health professionals. ***Trust*** • If an individual’s privacy requirements are not met it may lead to a reduction of trust.	0.7
[29], Canada, March to April 2005	Survey, response frequencies	General medical/general public	1230	**Age, years** 480 (39), 18–39 504 (41), 40–59 246 (20), ≥ 60 **Education** 406 (33), HS or less 172 (14), some post-secondary 492 (40), completed post-secondary 123 (10), post-graduate or professional degree **Sex, male** 554 (45)	Privacy Trust	***Privacy*** • Protection of privacy of their personal information was very important (74%) to respondents. • 56% increased concern about privacy in the past 5 years. • 68% agreed with the statement: ‘research that could be beneficial to people’s health is more important than protecting people’s privacy’. ***Trust*** • High levels of trust (26–35%) in disease-based foundations, hospitals, university researchers, and data collection organisations. • High levels of distrust in the insurance industry (42%), drug companies (28%), and government (27%).	1
**Qualitative**							
[30], United Kingdom, not reported	Interviews, thematic analysis was undertaken using the Framework approach	General medical/individuals included in the ALSPAC birth cohort study	55	**Age, years** 12 (21.8), 17 35 (63.6), 18 8 (14.5), 19 **Education** 7 (12.7), at university 25 (45.5), A-levels 8 (14.5), GCSE’s 12 (12.8), other 3 (5.45), none **Health status, self-reported** 9 (16.4), disability/long-term illness 46 (83.6), no disability/long-term illness **Sex, male** 24 (43.6)	Privacy Transparency Trust	***Privacy*** • Data linkage best practice provides enough privacy protection. • Threat to privacy was a potential harm. • Data anonymisation was a solution to privacy concerns. ***Transparency*** • Data linkage processes should be clear and transparent. ***Trust*** • A lack of trust about how data would be used. • Trust was associated with the need for consent. • Important that research does not undermine an individual’s trust in research.	0.95
[31], England, Wales, and Scotland (UK), March to April 2008	Face to face interviews, adjusted proportions	National cancer database/ general public	2872	**Age, years** 1315 (46), 16–44 997 (35), 45–64 564 (20), ≥65 **Education** 542 (19), Degree or higher 1496 (52), Below degree 837 (29), No qualifications **Ever had cancer? No** 2701 (94) **Sex, male** 1319 (46)	Privacy	***Privacy*** • 81% would support legislation to underpin the National Cancer Registry. • 95% did not believe that a letter from their primary care trust inviting them to a cancer screening test was an invasion of privacy. • 80% did not consider the inclusion of their postcode or name and address in the registry, or the receipt of a letter inviting them to take part in a research study, was an invasion of their privacy. • 2% saw all three scenarios as an invasion of privacy. • Small but significant differences were found according to country, ethnicity, socioeconomic status, housing tenure, and the experience of cancer in the immediate family. • The majority did not consider the confidential use of personal, identifiable information by the National Cancer Registry for the purposes of public health research and surveillance to be an invasion of privacy. • The confidential use of identifiable health information for research without individuals’ consent has not damaged the public’s trust so far.	0.95
[32], Belgium, February 2017	Interviews, deductive analysis using QUAGOL	Reuse of clinical trial samples and data/ clinical trial participants	16	**Age, years** 35–79, mean 62, median 64 **Sex, male** 7 (43.75) **Education** 10 (62.5), higher education 6 (37.5), college or university **Ethnicity** 15 (93.5), Belgium 1 (6.25) Polish **Cancer types** 4 (25), colorectal 3 (18.75), ovarian 1 (6.25), gastric and lung 1 (6.25), colorectal and lung 2 (12.5), pancreatic 2 (12.5), gastric 1 (6.25), cholangiocarcinoma 1 (6.25), unreported	Privacy Transparency Trust	*Only results about data sharing are reported* Data was seen by participants to be a similar resource to tissue samples; however, this position is not supported legally where the samples are not considered the same. ***Privacy*** 2 (6.25) explicitly specified that information provided to other research groups would be coded, illustrating a wish to protect their privacy. • If this is protected, then there was a willingness to share data for research. Participants supported medical research and the reuse of data for altruistic reasons. Most interviewed believed that the data can and should be reused. However, the participants should little interest in the specific purpose for reuse. • 2 (12.5) expressed a duty to contribute to science and wanted the maximum potential extracted from their data. The privacy and security of systems to exchange data is essential. ***Transparency*** Some participants wanted to be informed when their data was being used as this increases research transparency. Participants were mostly positive about digitisation in health as the use of these tools may increase control and transparency and allow for greater participation in research. ***Trust*** Participants were generally trusting of the initial research team. • 1 (6.25) participant noted that further research was acceptable, if it ‘stays within the oncology area’. Responses showed that participants trusted their clinicians to use the data correctly in their disease setting. General trust in the health services and clinicians. 14 () trusted the ethics committees to be the decision maker about access to data. *Distrust* • 2 (12.5) participant distrusted ‘unknown’ researchers, particularly the potential for data misuse and security. 1 (6.25) participant expressed his distrust in electronic systems generally as they become alternatives to traditional provision of care.	0.9
[33], England, September 2015 to December 2017	Interviews and online survey, thematic analysis	Human Fertilisation and Embryology Authority registry/fertility clinic attendees	60 (20, interview, 40, online survey)	*Interview population* **Age, years** 36 median, 30–46 range **Ethnicity** 16 (80, British white **Sex, male** **5 (25)** **Occupation** 14 (70), managerial or professional 2 (10), intermediate 3 (15) routine or manual 1 (5), student	Privacy Transparency Trust	*Interview population* 14 (70) agreed to share data 2 (10) refused to share their data 3 (15) were unsure about sharing data 1 (5) agreed and disagreed with data sharing at different times *Online survey* 32 (80), agreed to share data 4 (10), refused to share their data 2 (5), were unsure about sharing data 2 (5), agreed and disagreed with data sharing at different times **Privacy** Differences in sharing were seen by professional status, with those in intermediate or manual or routine jobs being more likely to share data (interview participants only). Sharing data for the greater good was important to some, however others believed that it may cause harm (fraud, identity theft, targeted for marketing). • Documentation needs to clearly say that the data will only be used for research and not marketing. • Concerned about harms not just to them, but also their children. • Not sharing is a mechanism to protect themselves. Stigma around IVF treatments. • HFEA data is required by legislation; however, some expressed concern that that the requirement to collect this type of data is not required in fertile couples. **Transparency** Many were not sure what they had already agreed to in respect to sharing data. Variation in the understanding of anonymisation and identifiable data. **Trust** Many already thought that data was already shared for research purposes. Trust in partners, clinics, hospitals, and wider institutions was noted. Misconception about the data being shared, some believed that it was NHS data which is generally seen as a trustworthy data custodian. • This trust was not universal as some had lots trust after recent data beaches by this organisation. Trust in the fertility clinic was reduced as they were seen as businesses.	0.9
[34], Scotland (UK), May to June 2009	Focus groups, thematic analysis	General medical/general public	19	**Age, years** 1 (5), <60 15 (79), 60–74 3 (16), ≥75 **Numbers taken part in medical research** 6 (32) **Numbers with chronic health condition** 13 (68) **Numbers with loyalty cards** 15 (79) **Sex, male** 6 (32)	Privacy Trust	***Privacy*** • Self-worth was articulated as a question of rights (i.e. ‘Isn’t there a right to privacy though?’). • Beliefs that an individual has a ‘natural right to privacy’ were balanced with a genuine commitment to supporting medical research. • AS belief that the institutions and researchers to keep their medical records private and confidential, was associated with a decreased requirement for consent. ***Trust*** • Respondents acutely aware of previous information security breaches, however continued to display a high level of trust in the organisations staff. • Trust was highest for clinicians involved in a person’s care but was extended to researchers more generally. • Concerns about control and access and a generalised scepticism and mistrust of the government and large commercial and insurance organisations.	0.85
[35], USA, March 1996 to February 2000	A single structured interview, Pearson’s chi-square test for independence, logistic regression models (binary responses) and ordinal logistic regression	General medical/individuals with genetic or chronic health condition* (or a family member with condition)	602	**Disease** Participants were equally divided (approximately) into the six diseases. BC and CC each comprised of 50 participants with a personal history of the disease and 50 with a family member with the disease.	Privacy Trust	***Privacy*** • 2% thought computerised records were a negative due to privacy violations. • Those who agreed with anonymised computer records, believed that it would advance medical research without violating privacy. ***Trust*** • Some did not trust that computer databases set up anonymously for research would be secure. • If individuals trust the entity asking to use the data, they may better trust that the records will remain confidential and will be used for worthwhile purposes. • Important that the integrity of medical care and research enterprise is maintained, and patients trust their physicians and medical institutions. • Institutions have a responsibility to take trust seriously, and never assume a simple right to conduct research with private, identifiable data.	1
(28), Australia, February to December 2006	Focus groups, thematic analysis Survey, chi-square test of independence	General medical/general public	723 (23, focus group 700, survey)	**Age, years (survey population)** 35 (5), 18–19 138 (19.7), 20–34 141 (20), 35–44 208 (29.7), 45–59 178 (25.4), ≥60 years **Education (survey population)** 66 (9.4), not finished HS 159 (22.7), finished HS but no HSC 131 (18.7), finished HS and HSC 17 (2.4), some technical or commercial/ TAFE 24 (3.4), finished technical or commercial/ TAFE 13 (1.8), some university/ C.A.E. 85 (12.1), tertiary diploma 15 (2.1), now at University/ C.A.E. 145 (20.7), university/C.A.E. degree 40 (5.7), post-graduate degree	Privacy	*Note: Results from survey population* ***Privacy*** • 98% supportive of general medical research; 73% would be happy to share their health data for research; 12% would not share their data; 14% were not sure. • 37% concerned about sharing their health information for research; 33% were not concerned. • The youngest and oldest age groups were less concerned about privacy (51.4% and 53.4% respectively) compared to the other age groups (range between 71% and 76%). • Those with poorer health were less concerned about privacy compared to those in good health (63.8% vs 67.7%). Of those who did not comment on health status 93.3% had concerns about privacy. • Even with additional security measures and anonymised data, 25% remained concerned about privacy. • Those not employed were less privacy concerned (60%) than respondents who were employed (range 66–74%). • Most concerns about data sharing related to: sexually transmitted diseases; issues of abortion, infertility; family medical history/genetic disorders; drug/alcohol incidents; mental illness; list of previous operations/procedures/dates; and current medications. • For cancer: 12% were very concerned, 15.7% were concerned, 9.7% were slightly concerned, while 62.3% were not concerned. • Most want to be asked for their permission before their health information is used for any purpose other than medical treatment (92%), and they would like to know the organisation and details of the research before allowing the use of their health records (83%). • Many not aware that removing names from medical records does not guarantee confidentiality. • Insurances that security measures protect confidentiality of personal health information are in place are important, as are reassurances that this will be done consistently and reliably.	0.95
[36], USA, not reported	Focus groups, grounded theory approach	Arthritis and other chronic conditions/hospital	23	**Age, years, mean** 59 (SD ±13), range 36–84 **Sex, male** 5 (21) **Chronic Illness** 10 (43.5)	Privacy Trust	***Privacy*** • Confidentiality was noted as a concern generally for patients. ***Trust*** • Trust, distrust, and confidentiality were influential and a consideration in patients’ views towards research registry participation. • A trusting doctor-patient relationship might be an important factor influencing registry participation.	0.95
[37], USA, not reported	Focus groups, emergent content analysis	General medicine/general health	30	**Age, years** 1 (3), 18–30 4 (13, 31–40 4 (13), 41–50 8 (27), 51–60 4 (13), 61–70 6 (20), 71–80 1 (3), ≥80 **Sex, male** 14 (47) **Education** 11 (37), some HS 7 (23), HS 7 (23), some college 3 (10), college **Ethnicity** 4 (13), white 5 (17), black 20 (67), Latino 2 (7), other	Privacy Transparency Trust	***Privacy*** • Some respondents were concerned about the loss of privacy. • Respondents preferred that their electronic data be accessed over paper-based records. • Concerns regarding privacy violations revealing highly personal information and third-party access to data were expressed. • If no permission to use data sought, some considered it an invasion of their privacy. • Using the data to support research recruitment was seen a violation of privacy. ***Transparency*** Development of transparent policies and practices will continue to support the secondary use of health data. ***Trust*** • There is a psychological component of uncertainty and mistrust. • Lack of trust regarding volunteering for research. • Participants wanted to have face to face contact with researchers during the recruitment process to increase trustworthiness. • Research organisations and institutional review boards need to develop outreach programmes to engage communities and build trust.	1
[38], USA, 2006 and 2008	Interview (telephone and enhanced face to face), multilevel random effects logistic regression	General medicine/health and retirement study	6384	**Age, by birth cohort** 747 (11.7), <1923 428 (6.7), 1923–1930 3543 (55.5), 1931–1941 792 (12.4), 1942–1947 875 (13.7), 1948–1953 **Sex, male** 2522 (39.5) **Ethnicity** 5235 (82.0), white 875 (13.7), black 275 (4.3), other **Education** 1481 (23.2), 0–11 years 2190 (34.3), 12 years 1334 (20.9), 13–15 years 1379 (21.6), ≥16 years	Privacy	***Privacy*** • Concerns regarding privacy and confidentiality were reflected in rates of consent to data linkage (LR 43.48, *p* < .01) • As the rate of concerns regarding privacy and confidentiality increases, lower rates of consent are seen. • Where respondents refused to answer questions regarding their finances, consent for data linkage decreases. The hypothesis that privacy and confidentiality concerns are negatively related to consent was supported.	1
[39], Australia, not reported	Interviews, framework approach	General medicine/general public	26	**Age, years** Between 24 and 41 **Education** 3 (12), ≤ Year 12 6 (23), TAFE 16 (62), tertiary 1 (4), post-graduate **Sex, male** 6 (23)	Privacy Trust	***Privacy*** • The protection of privacy was reflected in the need for consent, without which privacy might be breached. • Some believed de-identified information does not breach privacy. • Many not concerned about use of identifiable data to create the linkage key. This may indicate that views on privacy are changing. • Conflicting values, notably between privacy protection and public benefits. ***Trust*** • Concerns about identifiability when the linkage being done by researchers; not a lack of trust in researchers. Some concerns about the potential for a person known to them to find out private information. • Lack of trust that information would remain anonymous.	0.9
**Mixed methods**							
[40], USA, November 2003 to June 2004	Deliberative sessions and surveys Quantitative - chi-square for categorical data and ANOVA for continuous variables Qualitative-content analysis	General medical/veterans	217	*Characteristics of deliberators provided* **Age, years—mean (SD)** 65 (12) **Education** 80 (37), BS or BA or higher **Sex, male** 206 (95)	Privacy Transparency Trust	*Note: Results from deliberators provided* ***Privacy*** • Compared to university researchers (75%), participants were less inclined to give permission for a local hospital to use their medical records for a preventive health programme (61%) and even fewer (51%) were inclined to give permission to a drug company for marketing purposes. • Concern regarding the protection of privacy, particularly if enough was being done to protect their privacy. • Many conformable with using their records for research if their information stayed within the VA. • Participants wanted to know what data was being used and for what purposes; particularly how this information would be used to help other veterans. • 75% were unaware that in the US medical records could be used without permission. • Concerns regarding access to information about stigmatising conditions were raised, particularly HIV/ AIDS or mental health illnesses. • Concerned about the potential of their data being sold. ***Transparency*** • Even those with a high level of trust in the VA, want to be fully informed about how their records were being used for research and any conclusions from this research. • Ensuring medical records are being kept private and confidential in a way that patients can see is important. ***Trust*** • At follow up survey (4–6 weeks after baseline): 32% believed that medical researchers at a VA hospital would always keep their information confidential and private (change from 37% at baseline); compared to 8% for health insurance company researchers and 9% for pharmaceutical company researchers. • Respondents who trust the VA were likely to recommend a less-stringent process for obtaining consent.	0.9
[41], England, June to July 2016	Surveys and interviews, not described	Cancer registry/cancer patients and non-cancer patients, cancer	2033 (1033 with cancer, 1000 general public)	**Age, years, cancer group** 31 (3), 18–34 155 (15), 35–54 847 (82), ≥55 **Age, years, general public** 290 (29), 18–34 350 (35), 35–54 350 (35), ≥55 **Cancer status, cancer group only** 186 (18), localised/stable 31 (3), advanced 671 (65), remission/ cancer free **Cancer type, cancer group only** 52 (5), bladder 93 (9), bowel/colorectal 258 (25), breast 134 (13), prostate 62 (6), cervical/womb 155 (15), skin 300 (29), all others **Family or friend who has/had cancer, general public group only** 640 (64), yes **Sex, male** 475 (46), cancer group 490 (49), general public	Privacy Trust	***Privacy*** • Privacy concerns: 12% in the cancer group and 9% in the general public group. • Practical aspects of privacy: identifiable information being included (6% in the cancer group; 2% in the general public group) and concern over third parties having access to data (6% in the cancer group and 2% of general public). • Respondents with cancer who oppose the current method of collection, but that support collection of cancer data in general were mostly being put off by the lack of consent and lack of information rather than privacy concerns. • The trade-off between data collection for improved cancer services and treatment versus no data collection to maintain privacy and security; support for this trade-off was high across both groups. ***Trust*** • Concern regarding trust in organisations to maintain data security was noted in a small group of respondents.	0.6
[42], Australia, not reported	Focus groups and semi-structured interviews, open coding and NVivo analysis	Epidemiological research/general public and expert stakeholders	45 *(calculated based on the below)* (4 focus groups with general public (4 to 8 persons per group) 2 focus groups with Aboriginal and Torres Strait Islander peoples (4 to 8 persons per group) 5 people from diverse cultural backgrounds 20 expert stakeholders)	Not reported	Privacy Transparency Trust	***Privacy*** • Respondents wanted to know where their names and contact details were sourced for studies. ***Transparency*** • Wanted to know the source of funds for research, indicating a desire for transparency and the ability to judge the merits and motives for the research, to know who has vested interests in its findings. • Concern about a lack of transparency from research undertaken by pharmaceutical companies (for example not publishing adverse results). ***Trust*** • Prepared to participate in epidemiological research, particularly if it is conducted by a trusted public institution (government health departments, charities, universities). • Trust is critical to the conduct of research and it is important that research institutions do not act in ways that betray trust. • Widespread community distrust of research conducted or sponsored by pharmaceutical companies and private companies. • Pharmaceutical companies were repeatedly singled out by participants – concerns regarding their motivations were raised (i.e. profits).	0.9
[43], England, April to May 2013	Focus groups and interviews not described	General medical/general public	50	**Age, years** 18–70	Privacy Transparency	*Note: Telephone interviewees were defined as the ‘pro-privacy group’ and were respondents who were especially cautious about releasing personal data and actively taking some measures to protect against doing so. The focus group respondents were recruited as owners of products such as a loyalty store card.* ***Privacy*** • Respondents from the telephone interview group were more fearful of ‘what ifs’ and were cautious in releasing personal data because they worked in industries responsible for handling personal data (e.g. banking, insurance, selling databases). • Concerns regarding the potential for data to be lost, stolen, hacked, or leaked, and shared without consent. • Invasion of privacy, with a sense of Big Brother watching; incorrect or inaccurate data collection, which would be hard to correct and undo; potential discrimination (e.g. data falling into the hands of an employer). • Strong feeling that personal health data are confidential, private, and sensitive, and should not be shared outside secure, authorised bodies, and especially not with private companies such as employers, insurance providers and drug manufacturers. • Mental health data was sometimes regarded as particularly personal and sensitive. • Some concern regarding the potential for future discrimination being introduced, or was already being practised, within the NHS (e.g. low priority on waiting list; being refused treatment until lifestyle changes are made). • Concerns regarding potential privatisation; health data may be vulnerable to outside parties. • Possibility of individual identification was a cause for concern. • Fears about research/clinical trial data were low and related to anonymity being lost and possible unwanted media attention. • Data linkage was more acceptable at aggregate level rather than individual level. ***Transparency*** • Clarity, transparency, and reassurance required when addressing issues of linking and use of personal data.	0.9
[44], Great Britain, November to December 2015	Deliberative workshops and face to face interview, not reported	Commercial access to health data/general public, doctors, individuals with chronic or rare disease	2263 (246 focus groups 2017 interviews)	Not reported	Privacy Transparency Trust	***Privacy*** • Clear benefit both to individuals and to wider society was the only ‘good’ rationale for breaching privacy. • Different data types came have different privacy expectations. • New ways of collecting and sharing data may give rise to conflicting expectations around data privacy. • Concepts of privacy are in flux. • Privacy should be the default position and that any sharing would infringe that privacy. • Data anonymisation was a potential safeguard. • Permission for a commercial access was noted as a concern some respondents feel that they are somehow losing their privacy. • Respondents with pessimistic dystopian outlooks were not only concerned about the potential negative effect on themselves but fear large-scale negative impact for all society. ***Transparency*** • Underlying concerns for equity, transparency, and independent scrutiny of research by bodies free from vested interest. Restricting third-party access, and transparency in sharing results and publishing analysis. • Transparency was universally important. ***Trust*** • Motivations of commercial companies were questioned, and the private sector was mistrusted in general. • When benefits are perceived (and the organisation is trusted) all participants accepted commercial access to health data in principle. • The nature of the safeguards is not as important as the trust that comes with knowing there is a safeguard in place. • Education on aggregation and anonymisation is required to build public trust. • 20% of respondents said commercial organisations cannot be trusted to store the data safely, 18% say that profit should not be made from their NHS health data, even if there are potential societal and health benefits; 13% fear that a commercial organisation might sell the data on to another commercial organisation. • Some lacked understanding of, or trust in, anonymisation, and also did not know how data is actually held in the health service. • Access to data by insurance company to adjust premiums was met with universal disapproval. • If respondents did not trust the organisation conducting the research, they called into question the ultimate public benefit. • Only allowing named individuals within an organisation to access health data has had little traction – the individual’s may not be trustworthy. • Some have limited trust in commercial organisations undertaking research and not only fear no public benefit but allowing access to health data will create new public harms.	0.95
[45], Canada, not reported	Public dialogues and survey, ANOVA and MANOVA	General medicine/general public	98	**Age, years** 37 (38), 20–39 35 (36), 40–59 26 (27), ≥60 **Education** 26 (26), HS or less 18 (18.7), some post-secondary 42 (42.7), completed post-secondary 12 (12.5), post-graduate or professional degree **Sex, male** 40 (40.8)	Privacy Trust	***Privacy*** • Providing information about research allows individuals to feel like they are contributing while respecting privacy. • Some respondents were less privacy concerned. ***Trust*** • Accountable systems for managing data can increase trustworthiness.	0.5
[46], Canada, November 2006 to July 2007 and September 2007	Survey and focus groups, regression analysis using generalised estimating equations	General medicine//DM, HT, chronic depression, alcoholism, HIV, BC, LC, and general public.	1780	*(n = 1137)* **Age, mean** 54 years **Sex, male** 765 (43) **Education** (33), HS or less **Self-described health** 587 (33), poor or very poor 712 (40), fair 481 (27), good or excellent	Privacy Trust	*Completed survey n = 403* ***Privacy*** Respondents who were more privacy sensitive were less inclined to participate in the study. Disclosure concerns differed across health conditions. Respondents who completed the survey via telephone were less privacy concerned compared to those who completed it online. ***Trust*** Participants trusted that their information would be used appropriately. Higher levels of trust were placed in the individual (doctors) (64%), as well as hospitals (43%); this was higher than for university researchers (28%). Lowest rates of trust were recorded for: pharmaceutical companies (9%), provincial governments (9%), and insurance industries (6%). Research undertaken for profit or linking of income, education or occupation had lower rates of consent. Trust is reduced in the event of high-profile data breaches.	0.9
**Other**							
[47], New Zealand, not reported	Citizens’ jury, not applicable	Pharmaco-epidemiological research/general public	13	**Age, years** 18–65 years (7 (54) were 45) **Sex, male** 6 (46)	Privacy	***Privacy*** • Given appropriate privacy safeguards, an informed public does not always place personal privacy above societal benefits. • A balance between the public interest and medicine safety is important. • The use and linkage of medical information for research on medicine safety is warranted given the existing protections and the minimum of identifiable information. The jury was comfortable with the small loss of privacy to support public good and safety…	1
[48], United Kingdom, not reported	Citizens’ jury, not applicable	General medicine/general public	34	**Age, years** 8 (23.5), 18–29 10 (29.4), 30–44 10 (29.4), 45–59 6 (17.6), ≥60 **Sex, male** 17 (50) **Education** 13 (38.2), no qualification 11 (32.4), apprenticeship or other qualification 10 (29.4), degree level or above	Privacy Transparency Trust	***Privacy*** • Public benefit was a key justification for access. • Where data was used for public benefit (such as improved medical care and treatments, improved public health, or management of public funds) and organisations made a clear and compelling case for why they need these patient records, access should be granted. • An individual’s right to privacy should not prevent research that can benefit the general public. *Privacy statements:* a. We should share all the data we can because it benefits the services and me—as long as I can opt out if I choose b. We should not share data as the risks to people’s privacy and security outweigh the benefits *19 (55.9), agree more with a than b.* *11 (32.4),* agree more with b *than* with a. 4 (11.8), agree equally with both or don’t agree with either or don’t know. ***Transparency*** • Transparency about data use and access was important. • Involving individuals in the decision regarding data use would increase transparency. ***Trust*** • Important to show a clear, relevant connection between the research question and the information contained in the records. Some organisations had a track record of protecting data and records and could be trusted to maintain control of data. • Some believed that organisations could not be trusted to maintain records appropriately. • Organisations may use the data to exploit or manipulate individuals or populations or might manipulate the data to support their own agenda. • Providing more information on data use does not increase public trust.	0.8

*AIDS* acquired immune deficiency syndrome, *ALSPAC* Avon Longitudinal Study of Parents and Children, *BC* breast cancer, *BHPS* British Household Panel Survey, *CAE* Centre for Adult Education, *CATI* computer assisted telephone interviewing, *CF* cystic fibrosis, *CC* colon cancer, *DM* diabetes mellitus, *ED* emergency department, *EU* European Union, *GCSES* General Certificate of Secondary Education, *GED* general educational development, *HFEA* Human Fertilisation and Embryology Authority, *HIV* human immunodeficiency virus, *HKID* Hong Kong Identity Card number, *HS* high school, *HSC* high school certificate, *HT* hypertension, *LC* lung cancer; *LR* likelihood ratio, *MS* multiple sclerosis, *NHI* National Health Index, *NHS* National Health Service, *NILT* Northern Island Life and Times, *NZ* New Zealand, *SCD* sickle cell disease, *PS* primary school, *QUAGOL* Qualitative Analysis Guide of Leuven, *SS* secondary school, *TAFE* Technical and Further Education, *VA* Veterans Affairs, *UK* United Kingdom, *USA* United States of America

*Adults or parents of children with CF, or adults or parents of children with SCD, or adults or parents of children with DM, or adults with HIV, or adults with BC, or adults with CC

### Study design, location, clinical focus, and study populations

Qualitative research methodologies included face-to-face interviews and/or focus groups [32–34, 36–38, 49]. Other designs included surveys [16–21, 23–29, 35, 39, 41, 44] and combinations of deliberative sessions with surveys [15, 40, 45, 46] and focus groups and interviews [43]. Two studies used a citizens’ jury model [47, 48] and another was a nested cohort within a randomised controlled trial [22]. Studies were conducted in several countries; a breakdown by country is presented in Table 3.

**Table 3:** Studies by country

Country study undertaken (in alphabetical order)	Number of studies	Reference
Australia	3	[39, 42, 49]
Belgium	1	[32]
Canada	5	[20, 23, 29, 45, 46]
England	1	[33]
Europe	3	[16, 17, 25]
Finland	1	[18]
Hong Kong	1	[22]
Great Britain	1	[15]
Japan	1	[19]
New Zealand	2	[28, 47]
Northern Ireland	1	[26]
United Kingdom	8	[27, 30, 31, 34, 41, 43, 44, 48]
United States of America	7	[21, 24, 35–38, 40]

Most articles focused on the general public’s attitudes towards secondary data usage, particularly in general medicine [18, 22, 25–30, 34, 37–39, 43–45, 48, 49], but also national cancer databases [31, 41], clinical trials [21, 32], fertility [33], pharmaco-epidemiological [47], and epidemiological [42] research. Other studies focused on health consumers’ attitudes to secondary data usage in individuals: attending US Veterans Affairs (VA) facilities [40] or recently discharged from tertiary care [15], or with arthritis and other chronic conditions [36]. Others were in the setting of human immunodeficiency virus (HIV) [49], breast cancer (BC), colon cancer (CC) [35], or heterogeneous cancers [19, 20], acquired immune deficiency syndrome (AIDS), or multiple sclerosis (MS), or mental health concerns [23], presenting with rare diseases [16, 17], in adults or parents of children with cystic fibrosis (CF), sickle cell disease (SCD), or diabetes mellitus (DM) [35], or in adults with potentially stigmatising health conditions (DM, hypertension, chronic depression, alcoholism, HIV, BC, or lung cancer) [46].

The majority of articles discussed general attitudes towards health data linkage and secondary use [16, 22, 27, 30, 37, 39, 43, 46], linking health administrative data to clinical trial data [20] or clinical trial data reuse [21, 32], linking administrative data to survey data [38], access to medical records [15, 19, 23, 25, 26, 28, 29, 34, 35, 40, 45, 47, 48], statistical databases [49], research registries [17, 18, 31, 33, 36, 41], and health data for epidemiological research [42]. Privacy as sociotechnical capital [24] and commercial access to health data [44] were considered in one article each.

### Study quality

Results of the quality assessment are provided in Table 2. QualSyst [12] scores ranged from 0.5 to 1.0 (possible range 0.0 to 1.0). While none were blinded studies, most provided clear information on respondent selection and data analysis methods and used justifiable study designs and methodologies. No key themes stood out for studies which received poorer judgements. No data were from randomised studies, with the highest level of evidence from a nested cohort study. Other data were obtained from lower-quality studies such as surveys and interviews.

### Themes

#### Trust

A total of 12,794 respondents provide a view on trust; results were from surveys, questionnaires, focus groups, and interviews. One study was a nested cohort in a randomised control trial and two used a citizens’ jury model. Participants emphasised that organisations must develop, maintain, and promote high levels of patient trust [24, 26, 42]. Developing this trust can be achieved through the maintenance of confidential records and by providing information on how the individual’s information is used and by whom [40]. The importance of trust in health organisations, clinicians, and university researchers was also noted [18, 26, 29, 40, 42], although generally respondents trusted that organisations would keep their data private and confidential and that this would not be intentionally violated [40]. If a personal connection with the research team is established, then it is easier for individuals to form a trusting relationship [23]. The highest levels of trust was placed in the doctor [16, 26, 46], the National Health Service (NHS) [26], and hospitals [16, 46], while the lowest trust was in commercial organisations [26], pharmaceutical companies and insurance companies [46], or for-profit organisations [16]. An individual’s trust in an organisation was a determinant of what level of control they preferred over their data [40] and their willingness to participate in research [42], with trust overcoming concerns about privacy and confidentiality [49]. Where an organisation shows clear and relevant connections between their research and the information contained in the records, respondents trusted that the organisations will maintain the data appropriately [48]. Ensuring researchers act in the patient’s best interest and clearly and transparently disclosing the research being undertaken also built trust [40]. Respondents were generally trusting of the original research team and they trusted that they would use their data appropriately [32]. In a study about the use of fertility data, many respondents believed that registry data was already used for research purposes thus showing an established trust in the clinic, hospital, and wider health institutions [33].

The ability to maintain data security, privacy, confidentiality, and accurate records, change or delete incorrect data, and ensure that data would not be used to discriminate against an individual, all contributed to levels of trust [26]. Granting access to a small number of named individuals was not seen as a solution to resolving privacy concerns, as these individuals themselves may not be trustworthy [44]. Any research undertaken using secondary data analysis must not undermine or compromise an individual’s trust in medical research [30]. The level of respondents’ education influenced their view of trust, with a higher level of education being more trusting of their government and research institutions compared to those who finished their education earlier [16]. In the setting of fertility, most respondents were willing to share their data, suggesting trust in the organisation and registry [33].

#### Distrust

In contrast, the theme of distrust was noted in several articles representing a total of 6830 respondents and included data from questionnaires, surveys, focus groups, and a citizens’ jury. A general distrust in the health system, research, and sharing of health information [30, 36] was noted, with some respondents not trusting any organisation with their data [26] or the organisational capacity to maintain records appropriately [48]. While there was a desire to support the use of anonymised health data for research purposes, concerns regarding trust in the systems and data security remained [34, 40]. The provision of information on the source of research funding [40, 42] and data management systems [45] can increase transparency and trust, but providing more information on data use does not necessarily increase public trust [48]. In a study from the UK, some individuals with a ‘pessimistic dystopian’ mindset had limited trust in commercial organisations accessing health data, believing it would create new harms [44], with some suggesting that organisations may use the data inappropriately (exploit or manipulate individuals or populations or might manipulate the data to support their own agenda) [48]. Access to information by pharmaceutical companies and insurance agencies had lower levels of support, suggesting a distrust in these organisations. Older respondents (≥ 65 years of age) showed less trust in these organisations compared to younger respondents (≤ 25 years of age) [16]. Respondents who believed that data sharing had more negative than positive effects were more likely to have a college education [21]. Generally, these respondents believe that people could not be trusted and were concerned about data reidentification and information theft [21]. These low levels of trust were associated with a decreased willingness to share data with both for-profit and non-profit organisations alike [21]. Sharing data with an ‘unknown’ researcher was also associated with distrust; further, some believed that the increased digitisation of healthcare would lead to a decrease in the traditional provision of care [32]. In the setting of fertility, respondents’ levels of trust decreased given some respondents saw them as a business; it is essential that the information provided to people clearly state the purpose of data reuse and should note that it would not be used for purposes such as marketing [33]. Lucero et al. noted that there is a psychological component to uncertainty and mistrust. This leads to a distrust in volunteering for research and the need for organisations and ethics review boards to engage with communities to build trust [37]. To decrease distrust, respondents wanted to have face-to-face contact with researchers during a study’s recruitment process [37].

#### Privacy and confidentiality: differences according to demographic and health characteristics

A total of 44,366 respondents provide a view on privacy and confidentiality. Responses were obtained through surveys, deliberative workshops, dialogues and interviews, and questionnaires.

##### General concerns about privacy

Concerns about privacy and confidentiality were one reason for not sharing health data [16, 28, 29, 36, 37, 40, 44]. One study noted that the respondents’ concerns about privacy had increased over the past 5 years [29]. Concerns about the sense of ‘big brother’ and the potential for data to be used to discriminate [43] were expressed, with some consumers expressing a belief in the natural right (not dependent on law or custom) to privacy [34]. Where safeguards were in place to protect the data, most respondents in one study were willing to share their data, irrespective of the proposed data use [21, 32], except in the setting of litigation [21].

##### Demographic characteristics

The inclusion of an individual’s postcode, name or address, and receiving a letter inviting them to participate in research from the cancer registry was not considered to be a breach of privacy [31]. In a study of UK respondents, no substantial differences in privacy concerns were found according to sex or age; however, small but significant variations were noted by factors such as education, ethnicity, socioeconomic status, and an experience of cancer in the immediate family [31].

In other studies, the relationship between age and sex and concerns regarding trust and privacy were contradictory. Younger respondents expressed higher levels of trust in researchers and were more willing to let their data be used for research, but they also had high levels of privacy concerns [18]. Conversely, other studies noted that older respondents were more likely to agree to data linkage [22], while respondents aged 18 to 19 years and over 60 years had lower levels of privacy concerns compared to other groups [49].

Levels of concern about privacy were also influenced by the respondent’s level of education and employment. Those with commercial or technical qualifications had more concerns regarding privacy compared to all other education groups and those with a post-graduate degree had the fewest privacy concerns [49]. One article considered privacy in the context of sociotechnical capital, composed of awareness of privacy, attitudes towards the importance of privacy and data sharing, and confidence in the ability to maintain privacy [24]. Individuals with higher levels of education and income had higher rates of health privacy capital [24]. In a second study, respondents who were employed in manual, routine, or intermediate work were more likely to share their data compared to those in professional roles [33]. Respondents employed by a government organisation were more concerned about privacy [49]. Respondents who did not respond to finance questions had lower rates of consent for data linkage [38]. Other influences on privacy included social networks [24]. In one study, differences in rates of privacy concern between those who answered the survey online compared to those who answered via telephone were noted; those who answered by telephone were less privacy concerned [46]. Some differences in privacy concerns were noted by country. A study of European respondents found those based in Sweden, Slovenia, and Denmark were less concerned about privacy concerned compared to respondents from Lithuania [25].

##### Health status

Health status also impacted privacy concerns. Respondents in good health were more likely to agree to the use of data in healthcare registries compared to those in poor health [18]. Nevertheless, in the setting of no additional digital security measures (restricted access, etc.) being applied, individuals with poor health were less concerned about privacy compared to those in good health [49].

#### Sensitivity of data

A total of 3347 respondents provided a view on sensitivity of data. Individuals may consider some forms of medical data to be more sensitive than others. Data related to sexually transmitted diseases, family medical history including genetic disorders, drug and alcohol use [49], and mental illness [43, 49] raised the most privacy concerns, particularly the possibility of inappropriate data access [23, 49]. A UK report noted that ‘new ways of collecting and sharing data, under new circumstances, can give rise to conflicting expectations around data privacy’ [44] and that different types of data came with different privacy expectations [44]. In one study, respondents believed that data was a similar resource as tissue samples, suggesting that data is equally as sensitive as biospecimens [32]. A study of respondents who were seeking fertility services found that while there was a willingness to share data, they were concerned about the potential for the data to cause harm (potential for stigma), not only to them but also their children [33]. Further, some respondents were concerned that the data collected on them, while required by legislation, was not collected on fertile couples [33].

#### Control of data

A total of 6859 respondents provided a view on control of data; results were obtained from surveys, a nested cohort, focus groups, and a citizens’ jury. Individuals’ desire to maintain some control over their health data was evident across studies, with many seeing this as key to transparency [40, 48]. Respondents were selective about those with whom they were willing to share their data. Respondents to two UK studies preferred their data to stay within the NHS [34, 43], with some believing that once data left the organisation control would be lost [34]. Health data access by private/commercial organisations [21, 26, 37, 41–44], pharmaceutical companies [21, 42, 43], and health insurance companies [21, 43, 44], all were seen as inappropriate. Respondents were concerned that insurance companies would use health data to adjust premiums which was considered inappropriate and without clear public benefit [44]. Not allowing third parties to access their data was based on a distrust of these organisations [42, 44], perceived lack of transparency from research conducted by pharmaceutical companies [42], concern about the companies’ motivations (e.g. profit, marketing) [40, 42, 44], doubts about data security, distrust in their capacity to put society before profit, and a belief that a commercial organisation may on-sell their data [44]. In one study, some respondents indicated they would prefer that research not be undertaken if it required allowing commercial access to health data; however, most respondents wanted third-party access to health data if disallowing this resulted in research not being undertaken [44]. In contrast, other studies found respondents were happy to allow pharmaceutical company access to their registry data if it is undertaken in a transparent manner [17], and where consent is sought, respondents in a second study accepted the principle of commercial access to health data [44]. Respondents to one survey suggested that increased sharing may be used for marketing purposes, stolen, used for profit, or to discriminate against an individual [21]. Further, some respondents wanted to be informed when their data was being used [32]. Digitisation of health data was seen by some as a mechanism to increase control and transparency over their data, and increased participation in research [32]. Some believed that consent was required to access records for research, or to identify potential research participants, without which it was a violation of their privacy [37].

#### Benefit to society

The importance of research and its benefit to society was noted as important in several studies with a total of 7006 respondents. It was noted that society’s views on privacy may be changing, creating conflicting values between privacy protection and public benefit [39]. Generally, respondents were positive about sharing their data for research [18, 20, 49]. In some circumstances, societal benefit may outweigh concerns regarding privacy [29, 44, 47]; further, research using health data should have a societal impact and not be undertaken just for academic reasons [40]. Ensuring transparency about the public benefit of research and sharing of results and analysis at a study’s conclusion [44] were important. Where data was used for public benefit, such as improved medical care and treatments, improved public health, or management of public funds, and organisations made a clear and compelling case for access to the data, access should be granted as it could potentially benefit both the individual and the health service [48]. Public benefit was seen as a justification for access to health data and an individual’s right to privacy should not prevent research that could benefit the general public [48]. Altruism was also noted as reason to support health research using existing data, with some wanting their data to be used to ‘maximum potential’ [32]. In the setting of fertility data, sharing for the greater good was important to some [33]; however, this was not universal as some believed that it increased the risk of harm (fraud, identity theft, targeted marketing) [33] and that the premise that public benefit outweighs privacy concerns was not supported [18]. In one survey, some respondents valued maintaining individual control over their data more than societal benefit and respondents expressed a lack of willingness to trade loss of privacy for public good [23]. This was echoed in a second study where the majority of respondents believed that the right to privacy should be respected over all else; however, respondents also believed that if the data were made anonymous and privacy was maintained, data should be used for research that benefits society [26].

### Views about specific data sharing scenarios

#### Digitisation of health records

##### Data linkage

A total of 700 respondents provided a view on health data linkage. An Australian study identified concerns relating to confidentiality during the data linkage process, specifically the possibility that the individual making the linkage may know the person and find out confidential information about them, although this was not universal [39]. The use of de-identified data was not seen as a breach of privacy [39] and that the current data linkage best practice provides sufficient privacy protection [30]. Transparency about process and data usage was an important factor in individuals’ decisions to allow data linkage [43]. The importance of ensuring privacy when undertaking the linkage of clinical trial data to health administrative data was seen as important [20]. A survey of UK respondents found that they were not concerned about health record linkage as long as the data was used to increase health knowledge, consistency between health services, and administrative efficiency [43].

##### Registries and patient-provided data

A total of 25,814 respondents provide a view on registries and patient-provided data. Studies indicated that use of health information exchanges and digital health platforms can improve care [25]. While these positives were noted, views on use of these technologies varied widely [36].

While respondents agreed with the principles of electronic data sharing, they desired transparency and a mechanism for independent scrutiny of data access and use [44]. Some respondents indicated a preference for more electronic health data sharing [25].

There was a high level of trust in using data from disease registries [17] and a willingness to share data; in a cancer setting, only a small number of respondents were opposed to data collection [41]. The most common concern about registry-based research was the protection of privacy [18]. One mechanism suggested to ensure maximum transparency in registry research was to involve patient organisations in the development of clinical trials and registries [17].

### Strategies to address privacy and trust concerns

#### Data security

A total of 25,052 respondents provided a view on data security. Many health organisations already have well-established protocols to ensure patient privacy. Transparent information about data security and protection measures is important to maintain trust [26, 32] with some suggesting that systems to protect confidentiality should be more secure for shared data than those used in usual medical practice [40]. Some respondents were particularly concerned about unauthorised access to their data [40] particularly the chance for data to be lost, stolen, or ‘hacked’, or shared without consent [43], although trust in researchers and health care providers to maintain data security [25] remained. Some respondents in one study were happy to ‘trade-off’ any potential risk to privacy and security for benefits, like improved treatment and services [41]. Breaches in data security significantly reduced the levels of trust in an organisation to keep the individual’s health data private [33, 46]. Interestingly in one study, respondents were more willing to allow access to their electronic records, compared to paper-based records [37]

#### The role of legal and ethics bodies in protecting privacy

A total of 4219 respondents provided a view on the role legal and ethical bodies have in protecting privacy. The use of laws, regulations, and policies to protect an individual’s privacy in the UK [31], the USA [40], and Australia [49] were noted. Without developing an understanding of individual privacy concerns and perceptions of privacy, King et al. note that it will be ‘impossible to provide adequate law as well as effective technical solutions for protecting privacy’ [49]. In the UK, laws allow for data use if the risk to privacy is proportionate [15]; the NHS *Code of Practice on Confidentiality* establishes rules for the protection of privacy [31]. In relation to new laws to improve data collection, one study noted that 81% of respondents (*N* = 2335) would support a law making a cancer registry statutory in the UK [31]. In the USA, mechanisms such as the National Institute of Health (NIH) requirement for manuscripts from NIH-funded research to be made publicly available were considered beneficial in fostering public accountability and trust [40]. Further, the US Health Insurance Portability and Accountability Act of 1996 (HIPAA) establishes a national standard for the protection of health information [40]. However, some believe that concerns about privacy are not fully addressed by HIPAA, which treats all health data, except psychotherapy, the same [40]. In a study, some respondents were unaware that under some circumstances their medical data could be used without their permission [40]. Respondents in two studies advocated for clear and consistently applied penalties for individuals who breach privacy, such as job termination, paying fines, and/or going to jail [40]; measures such as this may increase perceptions of trust and accountability [40]. The role of ethics and institutional review boards in protecting privacy was noted in two articles [17, 40]. Respondents supported the role of ethics committees to manage access to health data and trusted their decisions [32]. It is important that health consumers recognise the role of these bodies in regulating access to data for research [40] and in protecting patient rights [17]. Finally, the development of clear policies and procedures will allow for more support for the secondary use of data, while increasing transparency for the healthcare consumer [37].

#### Anonymisation

A total of 5302 respondents provided a view on data anonymisation. Data anonymisation was central to an individual’s decision to share health data for research or health and service improvement programmes [26, 28, 40, 49]. There was a lack of understanding between the terms anonymisation and identifiable data [33]. In one study, many respondents were in favour of anonymous databases for research, noting it was beneficial and would advance medical research without impacting on their privacy [35]. In the setting of appropriate privacy, confidentiality frameworks, and ethical oversight, Parkin and Paul note that an informed public are more likely to be receptive to research using potentially identifiable health information [47].

Even when data is de-identified, some respondents remained concerned in the setting of extra security measures and data anonymisation [49], which were not seen as safeguards [44]. Some respondents believed that even if data had identifying features removed it was not completely de-identified [26] and were concerned about sharing de-identified data with non-healthcare professionals [28]. Respondents were asked about their preferences for either a computer system or human programmer to anonymise (extract and link) data; some expressed concern about the potential for identification of individuals and noted a need for trust in the people undertaking these tasks [34]. While respondents recognised the capacity of computers to undertake the anonymisation process, they suggested they would not trust a completely computerised system citing concerns about data infrastructure and data accuracy [34].

#### Communication and education

A total of 8511 respondents provided a view on the importance of communication and the role of education in promoting data sharing. Providing increased information about data use and research more generally allowed individuals to feel their privacy is being maintained while contributing to health research with societal benefits [45]. Providing education was also seen as a mechanism to improve transparency [15, 16, 33] and trust [23, 26]. Specific information on how and when the data will be used [15, 45], and knowing how and where their contact details were sourced [42] were all important to individuals. Information on the data aggregation and anonymisation processes [44], and the systems used to protect data [40], should be provided. In a UK cancer registry study, cancer patients opposed to current data collection processes were more concerned about lack of information about the registry and consent processes than privacy [41]. Information and education about database governance, including data storage, length of data accessibility, and use of data, should be clear at the time of consent, particularly for data held in disease registries [17].

#### Consent

Most of the articles included in this subset discussed the connection between issues of privacy, trust, and transparency, and consent. Specific issues of consent in relation to secondary data use and sharing are discussed in a separate publication. Broadly, seeking consent for the secondary analysis of health data, either anonymised or potentially identifiable, was seen as a way to build trust, respect, and transparency [26, 30] and address an individual’s privacy concerns [23, 27, 39].

## Discussion

This systematic literature review highlights the ongoing complexity associated with secondary data analysis and linking health data. Data gaps identified included a paucity of information specifically related to our primary area of interest, and the attitudes of breast cancer patients towards the secondary use and sharing of health administrative and clinical trial data. Interestingly, given the high rate of cancer more generally in society, this population was underrepresented in the results.

While respondents believed that the principles of data sharing were sound, significant concerns regarding privacy, information security, trust, and transparency remain. Further, the diversity of attitudes towards privacy suggests that there is little clarity on what predicts an individual’s attitudes towards privacy, highlighting an area for future study. Many respondents supported the use of health data for social benefit; however, this was not universal. The literature underscores the importance of communication between those who collect data or act as data custodians and health consumers. Health consumers should be provided clear information on how their data privacy will be maintained, how the data will be secured, and how access to their data will be regulated. Providing increased information to health consumers about how, when, and where their health data may be used, and with whom it may be shared, is essential in the development and maintenance of transparent data sharing systems and policies. Concerns relating to privacy and the misuse of data may be, in part, mitigated by increased education of health consumers regarding their national privacy laws and regulations. Providing information on penalties for breaches of privacy and how an individual’s health data can and cannot be used is important. This may reduce some specific concerns regarding inappropriate use of data, ‘big brother’ sentiments, and any perceptions of discrimination based on data. While not specifically discussed in the articles, it is important to note that as the use of artificial intelligence increases in healthcare, ensuring penalties for discrimination based on data analysis will become more complex. Examples of discriminatory algorithms, in society and in healthcare have been highlighted by researchers [50–52], and these need to be closely examined and tested as our reliance on data-driven healthcare increases. Further, health consumers need to be provided information about how any research is undertaken including anonymisation and aggregation processes, and the requirement for ethics committee oversight.

Our results suggest that trust is an important component in the discussion regarding the secondary analysis of health data: trust in the organisations, clinicians, and infrastructures used to maintain data. Onora O’Neill has written extensively on issues of trust in a modern society and in health and argues that despite the sentiment expressed by some that trust more generally in society has decreased, it has not; rather the culture of suspicion has increased [53]. Therefore, it is essential that organisations wishing to undertake secondary analysis on their datasets need to develop trust between themselves and health consumers.

## Limitations

The papers included in this study were limited to those indexed on major databases; some literature on this topic may have been excluded if it was not identified during the ‘grey’ literature and hand searching phases. As the search was restricted to English language publications, some relevant literature may have been excluded from the search. Given the initial focus of this research being attitudes towards data sharing and reuse in breast cancer, individuals under 18 years of age were excluded from the analysis. A final limitation of this research is that much of the data was from research methods (surveys, interviews) that are not considered to be level 1 evidence; however, a randomised controlled trial methodology is not necessarily appropriate to this research subject.

## Implications

Results of this systematic literature review indicate that while respondents identified advantages in health information data sharing, including post-market medication surveillance and the potential to decrease medical errors, concerns relating to trust, transparency, and the protection of privacy remain. Additional work is therefore required within these areas during the conception, design, and implementation phases of any health data sharing programmes to ensure the balance between public benefit and individual privacy is maintained.

## Conclusion

The literature confirms that while consumers understand the benefits of health data sharing for research purposes, issues of trust, transparency, and privacy remain central to acceptance of health data sharing policies and programmes in the general community. Researchers and those undertaking secondary data analysis should work with consumer organisations to ensure consumer concerns are addressed.

## Data Availability

All data generated or analysed during this study are included in this published article.
